# Different Diode Laser Approaches in the Treatment of Oral Mucocele: Report of Two Cases

**DOI:** 10.1155/crid/7218406

**Published:** 2026-06-17

**Authors:** Leonardo Compagnucci, Maria Elena Grecolini, Cristiana Nocco, Giorgio Compagnucci, Andrea Abate, Valentina Lanteri, Alessandro Ugolini, Alessandro Bruni

**Affiliations:** ^1^ Private Practice, Tolentino, Italy; ^2^ Surgical, Medical and Dental Department of Morphological Sciences related to Transplant Oncology and Regenerative Medicine, University of Modena and Reggio Emilia, Modena, Italy, unimore.it; ^3^ Private Practice, Lecce, Italy; ^4^ Department of Sciences Integrated Surgical and Diagnostic, University of Genova, Genova, Italy, unige.it

**Keywords:** case report, diode laser, laser surgery, oral mucocele, salivary gland lesion

## Abstract

**Objective:**

To describe and compare two different diode laser approaches in the management of oral mucoceles of the lower lip.

**Case Presentation:**

Two patients presented with recurrent swellings of the lower lip, clinically diagnosed as mucoceles. Both lesions measured approximately 8–9 mm and had been present for several months, with repeated episodes of rupture and recurrence. In Case 1, a 40‐year‐old woman underwent complete excision using a diode laser. In Case 2, a young adult received a conservative diode laser technique involving removal of the cyst roof and laser ablation of the base.

**Results:**

In both cases, surgery was completed without complications. The excisional approach achieved rapid healing, excellent aesthetic outcome, and no recurrence at 1‐year follow‐up. The conservative technique resulted in a smaller surgical wound, uneventful healing, and similarly no recurrence after 1 year.

**Conclusion:**

Diode lasers represent a safe and effective option for the treatment of oral mucoceles, providing excellent intraoperative control, reduced morbidity, and high patient acceptance. These two cases highlight that both complete excision and conservative intralesional diode laser techniques can lead to successful outcomes without recurrence.

## 1. Introduction

Oral mucocele is a nonneoplastic cystic or pseudocystic lesion originating from major and minor salivary glands, and it is the most frequent lesion of the minor salivary glands, occurring at all intraoral sites except the hard palate [1]. The lesion is most commonly found on the lower lip (approximately 85% of cases), predominantly caused by repeated trauma such as lip biting or parafunctional habits, particularly in children and young adults [2–5]. Less frequently, mucoceles occur on the tongue, cheeks, floor of the mouth, and upper lip [2, 3, 6]. Although mucoceles typically arise from minor salivary glands, lesions occurring in the floor of the mouth are termed ranulas and originate from the major salivary glands, particularly the sublingual gland [7, 8].

The highest incidence is observed between 20 and 30 years of age (about 35%), followed by patients aged 10–19 years (24%), with no significant gender predisposition [2]. Histologically, mucoceles consist of cystic cavities filled with mucus, ranging in size from 1 mm to a few centimeters, with an average size of 5–9 mm in approximately 50% of cases, and exhibit slow development [9, 10]. The mucus often contains degenerated histiocytes, and the cyst lining is usually granulation tissue rather than epithelium [11].

Two etiopathogenetic types of mucocele are distinguished [12]. The most common is the mucous extravasation mucocele (EM, 80%–95%), caused by trauma‐induced rupture of the salivary duct with mucus extravasation into the surrounding connective tissue, eliciting an inflammatory response with macrophages and lymphocytes. EMs are pseudocysts without epithelial lining, typically localized to the lower lip, particularly in younger populations [13]. The less common retention mucocele (RM, 5%–15%) arises from obstruction of the excretory duct due to infection or sialolithiasis, leading to distension of acini and a true cyst lined by epithelium. RMs are more frequent in the elderly and in sites such as the floor of the mouth, cheeks, upper lip, palate, and maxillary sinus [9, 13, 14]. Trauma may cause RMs to convert to EMs by rupture. The pathological overlap between types and clinical distinction require further investigation [13, 14].

A recent dermoscopic study classified EM into three progressive types, from soft grayish nodules with branched reticular vessels, to erythematous nodules with vascular neoformations directed towards the lesion center [13]. Clinically, mucoceles present as single or multiple soft, smooth, dome‐shaped nodules, occasionally painful but often asymptomatic, with colors ranging from pink to translucent blue and white or yellow‐orange areas [15]. Ruptures may lead to temporary disappearance but recurrence is common, often delaying specialist consultation.

Accurate diagnosis is based on detailed case history and lesion examination. History of trauma or habitual lip biting, along with rapid lesion appearance and recurrence, strongly suggest mucocele. Imaging modalities such as X‐rays (for sialolithiasis), ultrasound, or MRI may be employed for precise localization and evaluation near vital structures like the labial artery [16]. On ultrasound, mucoceles appear as mucus‐filled cystic masses with fibroblast‐generated fibrillar processes [16]. Fine‐needle aspiration cytology may aid in diagnosis when salivary gland nodules or sizable masses are involved [17].

Differential diagnosis includes mixed glandular tumors, lipomas, fibromas, and vascular lesions such as angiomas, which should be carefully excluded due to bleeding risk during treatment [18, 19]. Fibromas are firm, mobile nodules arising from connective tissue hyperplasia, whereas mucoceles are more fluctuant and blueish. Biopsy is recommended for deep or suspicious lesions to rule out other neoplasms such as adenoma, cystadenolymphoma, neurofibroma, epithelioma, or cylindroma [17, 20]. Rarely, syphilitic gums or botryomycomas may present as pedunculated bleeding lesions at this site [17].

Mucocele treatment options include classic surgical excision, electrocautery, cryotherapy, and laser therapy. The lesion typically enlarges slowly, punctuated by episodes of rupture and transient deflation; spontaneous resolution is rare and recurrence common [19]. Surgical excision remains the treatment of choice, aiming for complete removal of the cyst and involved gland to minimize relapse [19]. Surgical techniques comprise complete excision, marsupialisation, or dissection, selected based on cyst size and anatomical context. Marsupialisation involves opening and maintaining the cyst cavity to promote healing and epithelialization, using packing agents to prevent premature closure [21]. Though marsupialisation has lower relapse than dissection or excision, it is more often used for ranulas [22].

Traditional scalpel surgery is limited by intraoperative bleeding impairing visibility, risk of damaging adjacent structures including nerves and arteries, postoperative pain, edema, scarring, paraesthesia, and longer healing times; these factors reduce acceptability, especially among pediatric patients [23–28]. Electrosurgery is infrequently used, while cryotherapy—though applicable without anesthesia—may induce nerve and ductal injury, particularly in ranulas.

Laser therapy, especially diode laser, has gained growing acceptance due to superior hemostatic control, precision, decreased postoperative pain, accelerated healing, and improved patient acceptance. Systematic reviews affirm laser efficacy in pediatric patients, reporting minimal bleeding, fewer complications, and reduced relapse rates compared to traditional surgery [29, 30]. Lasers provide improved operative control and are preferred by clinicians for their ease of use and favorable clinical outcomes [29]. Although accumulating evidence supports laser therapy as an increasingly recognized as an effective and reliable approach for oral mucocele treatment, further comparative trials are warranted. These case reports describe two different applications of diode laser surgery in the treatment of oral mucoceles. By comparing total excision and a conservative intralesional technique, they highlight individual considerations in procedure selection and provide practical insights into clinical management. These cases aim to illustrate the potential benefits and versatility of diode laser therapy in everyday practice, adding further experience to the existing literature.

## 2. Case Reports

The current report was prepared following the CARE guidelines for standardized clinical case reporting, ensuring comprehensive and transparent documentation of clinical findings, interventions, and outcomes [31]. This study reports individual clinical cases and did not require formal approval from an institutional ethics committee according to local regulations. All procedures were performed in accordance with the ethical standards of the Declaration of Helsinki. Written informed consent was obtained from all patients for treatment and for the publication of anonymized clinical data and images.

### 2.1. Case 1

**Figure 1:**
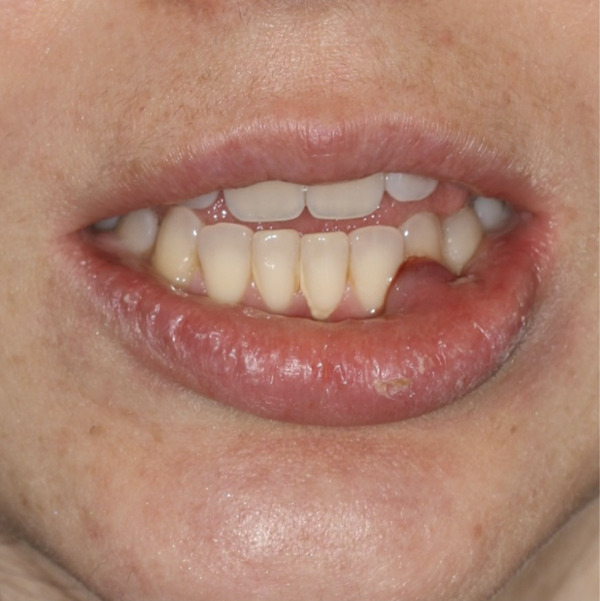
Case 1: extraoral view of the lower lip swelling.

A 40‐year‐old woman presented with a 6‐month history of a slowly enlarging swelling on the left lower hemilabium, which periodically ruptured and partially deflated but recurred with increasing volume after each episode (Figure 1).

**Figure 2:**
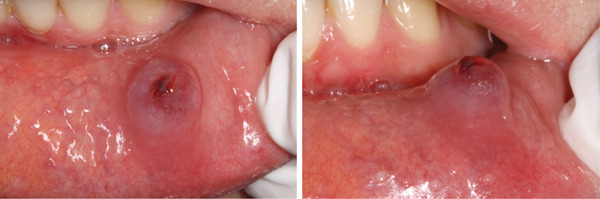
Case 1: intraoral view of the lesion from two perspectives.

**Figure 3:**
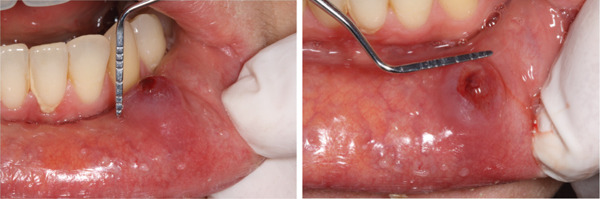
Case 1: further intraoral views of the lesion.

The patient denied relevant medical, family, or psychosocial history and reported no previous interventions for this lesion. Clinical examination revealed a well‐defined, soft, fluctuant, bluish‐pink nodule approximately 9 mm in diameter and 7 mm high, protruding into the oral vestibule and interfering with mastication due to frequent accidental trauma from biting (Figures 2 and 3). There was no associated pain, systemic symptoms, or evidence of infection. On palpation, the lesion was compressible and nonadherent to deeper structures.

**Figure 4:**
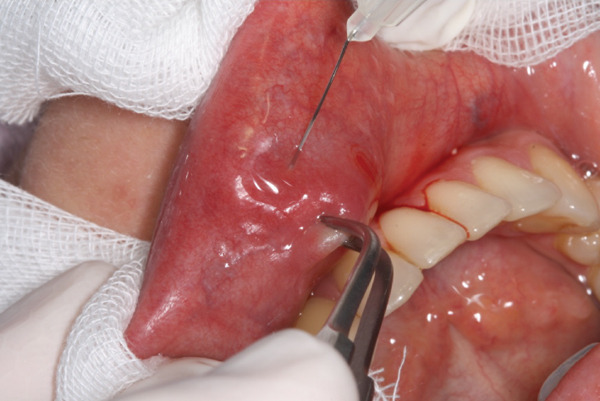
Case 1: initial diode laser incision.

No imaging was required as the diagnosis was clinically evident, supported by the lesion′s classical features and patient history. After informed consent, the therapeutic intervention was complete excision. The surgical excision was performed using a diode laser device (Fox III, a.R.C. Laser GmbH, Nürnberg, Germany) operating at a wavelength of 810 nm. The laser was used in continuous wave (CW) mode with a power output of 2 W, delivered through an optical fiber with a core diameter of 300 *μ*m. This configuration corresponds to a power density (irradiance) of approximately 2829 W/cm^2^ at the fiber tip. Because the laser was applied in CW mode and manually moved across the tissue boundaries, the cumulative fluence (J/cm^2^) depends directly on the overall exposure time and the moving speed of the fiber during the procedure. Following mild local anesthesia with articaine and a low concentration of adrenaline (1:200,000) administered at the base of the lip—to minimize swelling that could hinder visualization—the surgical procedure began with an initial laser incision of the lesion (Figure 4).

**Figure 5:**
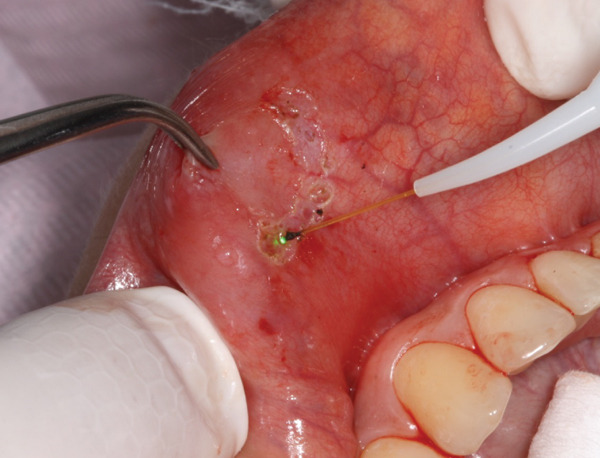
Case 1: intraoperative dissection with diode laser.

**Figure 6:**
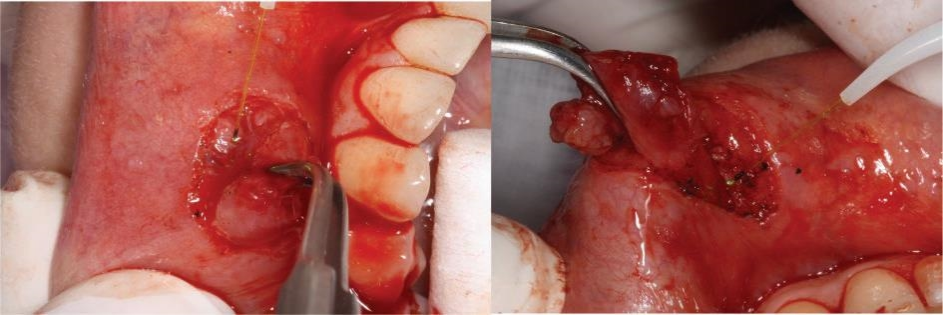
Case 1: intraoperative views from different angles .

**Figure 7:**
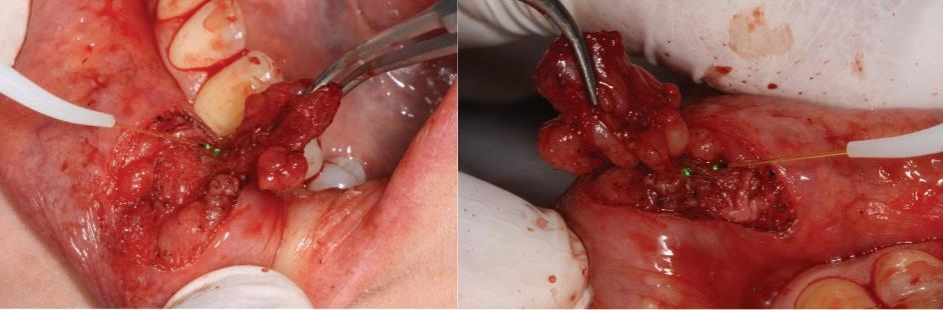
Case 1: progressive excision of the lesion.

Subsequently, the cyst was carefully grasped with an anatomical clamp and gently tractioned while the diode laser fiber was advanced underneath. This technique allowed progressive separation of the cystic lesion and the surrounding minor salivary gland lobules from adjacent connective tissues (Figures 5, 6 and 7).

During the dissection, special attention was paid to preserving critical anatomical structures, particularly the labial artery, which lies superficially at depths ranging from approximately 4 to 8 mm near the labial commissure, and to avoiding injury to nerve fibers to prevent sensory deficits. Because mucoceles are often pseudocysts—the cavity surrounded by proliferating granulation tissue containing extravasated saliva rather than a true epithelial lining—careful and precise dissection was essential to excise the lesion completely while minimizing trauma to surrounding tissues.

The use of diode laser technology provided effective hemostasis and enhanced surgical control, enabling precise enucleation of the cyst and associated glands, with reduced operative time and minimal postoperative morbidity.

The excised lesion measured approximately 15 × 10 mm and was immediately fixed in 10% formalin for histopathological examination. Microscopic analysis confirmed a cystic cavity lacking epithelial lining, surrounded by granulation tissue containing mucus and numerous foamy histiocytes, consistent with an extravasation‐type mucocele involving minor salivary gland tissue. The presence of adjacent glandular structures and the typical mucin content supported the diagnosis.

**Figure 8:**
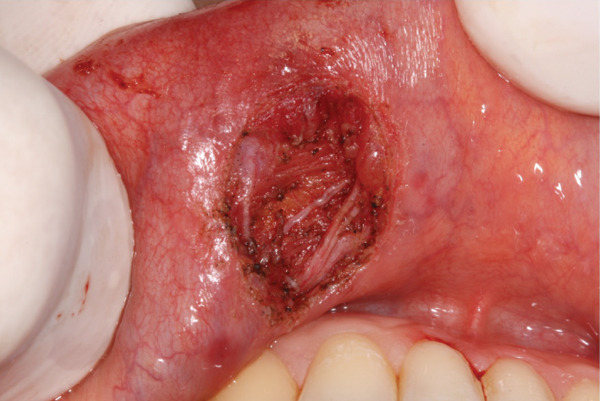
Case 1: immediate postoperative aspect after diode laser excision.

The intraoperative course was uneventful, with minimal bleeding due to the inherent coagulative properties of the diode laser, eliminating the need for sutures. The surgical wound exhibited immediate hemostasis and was protected by a thin coagulated tissue layer formed during laser cutting (Figure 8). Healing occurred by secondary intention, characterized by gradual contraction and mucosal reepithelialization.

**Figure 9:**
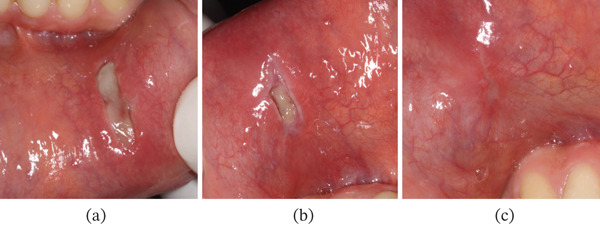
Case 1: postoperative healing at (a) Day 5, (b) Day 9, and (c) Day 18.

**Figure 10:**
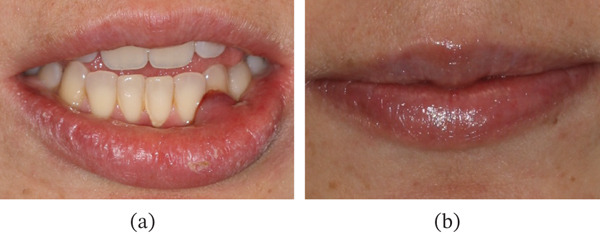
Case 1: (a, b) outcome after complete mucosal healing.

Postoperatively, the patient experienced an uneventful recovery free from pain, infection, or significant edema. Progressive healing was documented with notable wound size reduction by Day 5, near‐complete mucosal restoration by Days 9–18 (Figure 9a,b,c), and complete functional and cosmetic recovery thereafter (Figure 10). At 1‐year follow‐up, no signs of lesion recurrence or sensory disturbance were observed, and the patient expressed high satisfaction with the functional and esthetic outcome.

### 2.2. Case 2

**Figure 11:**
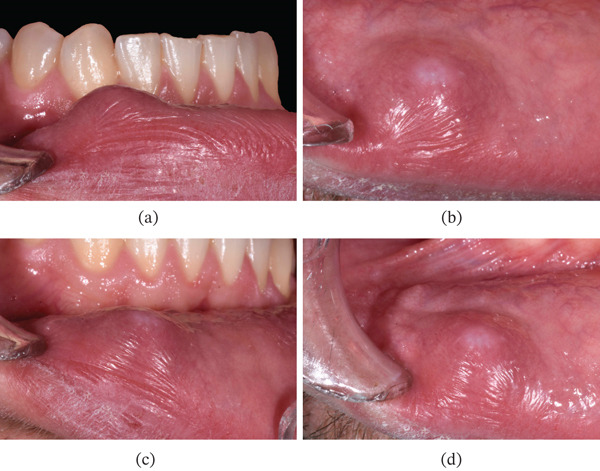
Case 2: (a–d) intraoral views of the lower lip lesion.

A 28‐year‐old male patient presented with a painless, dome‐shaped, bluish swelling on the central portion of the lower lip measuring approximately 8–9 mm in diameter and 5–6 mm in height (Figure 11).

The lesion had been progressively enlarging over the past 5 months with intermittent spontaneous ruptures resulting from accidental trauma during mastication, followed by rapid reaccumulation of the cystic content. The lesion was soft and fluctuant on palpation, freely movable beneath the mucosa, and caused mild local discomfort secondary to its frequent rupturing episodes. The patient′s medical, family, and psychosocial histories were unremarkable, with no prior interventions for this lesion.

**Figure 12:**
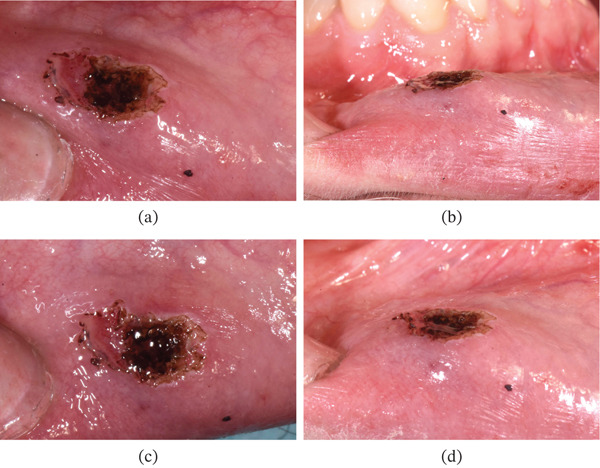
Case 2: (a–d) conservative diode laser procedure.

Based on the lesion′s clinical features and history, a diagnosis of oral mucocele was made. Given the patient′s young age and lesion accessibility, a conservative surgical approach using a diode laser was preferred. The procedure was performed using the same laser system and operating parameters detailed in Case 1 (810 nm wavelength, 2 W power output in CW mode, and a 300 *μ*m fiber, yielding a power density of approximately 2829 W/cm^2^). After administration of local anesthesia with articaine plus adrenaline (1:200,000) at the lesion site to ensure adequate analgesia and minimize tissue edema, the procedure entailed the removal of the cyst′s upper wall to evacuate its mucous contents, followed by the thorough laser ablation of the cyst base and the involved satellite salivary glands (Figure 12). This intralesional excision technique, distinct from classical marsupialization, allowed preservation of surrounding tissue while promoting healing from within the cystic space.

Due to the conservative nature of this technique, no specimen was available for histopathologic examination, representing a limitation in diagnostic confirmation. However, the minimally invasive approach resulted in a significantly smaller surgical wound compared to complete excision.

**Figure 13:**
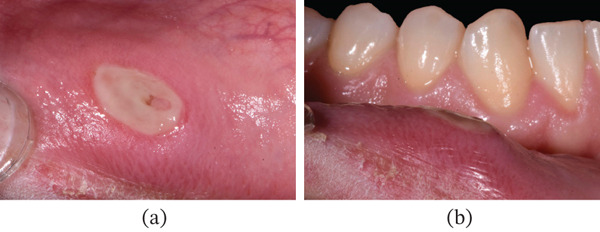
Case 2: postoperative healing at Day 5.

**Figure 14:**
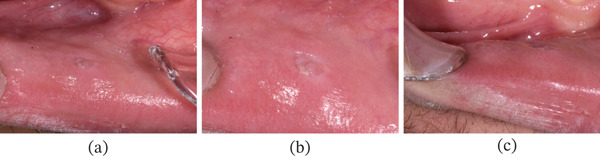
Case 2: postoperative healing at Day 14.

Postoperative healing was uneventful, with no pain, swelling, or functional limitations reported. The surgical site showed progressive epithelialization with marked wound reduction by Day 5 postprocedure (Figure 13) and near‐complete mucosal restoration by Day 14 (Figure 14).

**Figure 15:**
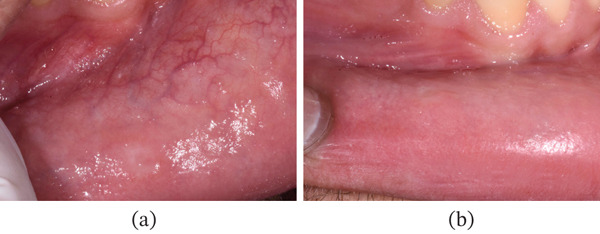
Case 2: one‐year follow‐up, normal mucosal appearance.

At 1‐year follow‐up, no recurrence was observed; the mucosa appeared normal and the patient expressed satisfaction with both functional and esthetic outcomes (Figure 15). No adverse events or complications occurred throughout the perioperative and follow‐up periods.

## 3. Discussion

These two cases demonstrate the effective application of diode laser technology in the surgical management of oral mucoceles, emphasizing its versatility and clinical advantages. Diode laser excision provides precise tissue ablation, effective hemostatic control, shorter operative time, and limited postoperative morbidity—advantages consistently supported by the literature. [19, 29]. The positive biological effects of laser applications on soft tissues, including modulation of inflammatory and angiogenic biomarkers, have been previously documented in periodontal setting also, supporting their broader use in oral lesion management [32].

The 810 nm diode laser has proven to be a highly versatile tool in oral soft tissue surgery. As documented in recent literature, diode lasers possess a high absorption affinity for chromophores such as hemoglobin and melanin, causing a localized elevation in temperature that promotes excellent coagulation and hemostasis [29, 33]. Beyond its application in mucoceles, the scientific literature extensively documents the efficacy of diode lasers for the excision of a wide range of other benign oral soft tissue lesions, including irritation fibromas and human papillomavirus (HPV)‐associated lesions (e.g., squamous papillomas) [34, 35]. When treating fibrous lesions such as fibromas, the diode laser allows for a rapid, bloodless excision with minimal thermal damage to surrounding healthy tissues, resulting in excellent esthetic outcomes, reduced postoperative discomfort, and minimal scar formation [29]. Similarly, in the excision of HPV lesions, the photothermal effect of the diode laser provides a distinct clinical advantage: it effectively seals blood and lymphatic vessels, providing a clean operative field that minimizes the risk of intraoperative viral dissemination while lowering recurrence rates [34, 35]. These benefits closely mirror the advantages observed in our mucocele cases, confirming the 810 nm diode laser as a reliable, minimally invasive alternative to the traditional scalpel for a wide spectrum of oral mucosal lesions.

In Case 1, complete excision with diode laser enabled the removal of the cyst and associated minor salivary gland tissue, facilitating histopathological confirmation and diagnostic certainty. This approach aligns with current recommendations emphasizing complete lesion excision to minimize recurrence and exclude malignancy [19, 30]. Rapid healing, minimal pain, and favorable cosmetic outcomes were observed [29].

Conversely, Case 2 exemplifies a conservative intralesional diode laser technique tailored to younger patients and smaller lesions. This technique minimizes surgical trauma and fosters rapid recovery, although it lacks histopathological confirmation due to the absence of excised tissue [19]. Comparable clinical success and low recurrence rates have been reported with conservative laser methods, particularly in pediatric and young adult populations [29, 30].

Both techniques offer superior patient tolerance, improved operative field control, and enhanced postoperative comfort compared to scalpel surgery [19]. The choice of surgical approach should be individualized according to lesion size, location, patient age, and diagnostic needs [29, 30].

For its ease of use, cost‐effectiveness, and large number of documented cases, lasers—especially diode lasers—represent a highly suitable technology for treating endo‐oral lesions, particularly mucoceles. Total excision with a diode laser ensures accurate tissue handling, minimal bleeding, and rapid, painless healing without complications. These features validate its use especially in pediatric patients and facilitate the clinical work of the operator [6].

The intralesional excision technique applied in the second case, which is not a classical marsupialization but an excision from within, results in even smaller postoperative wounds and rapid healing, enhancing patient comfort [3, 30].

Nevertheless, comprehensive statistical verification of techniques, healing times, and recurrence rates requires more numerous cases and systematic research [19, 30].

Diode laser therapy represents a reliable, minimally invasive, and highly adaptable approach for the treatment of oral mucoceles. In both cases presented, it ensured excellent intraoperative control, minimal bleeding, rapid healing, and high patient satisfaction, with no recurrence at 1‐year follow‐up. Compared to conventional scalpel surgery, diode lasers reduce operative time, postoperative discomfort, and esthetic impairment—features particularly relevant in young and pediatric patients.

Although current evidence and clinical experience strongly support its routine use, further prospective and comparative studies are needed to standardize operative protocols, define selection criteria between complete excision and conservative techniques, and evaluate long‐term recurrence rates. Overall, diode laser surgery should be considered a first‐line therapeutic option in the management of oral mucoceles.

## Author Contributions

**Leonardo Compagnucci:** investigation, resources, data curation. **Maria Elena Grecolini:** investigation, data curation, writing – original draft. **Alessandro Bruni:** conceptualization, methodology, investigation, formal analysis, writing – original draft, writing – review and editing, supervision. **Cristiana Nocco:** investigation, data curation, writing – original draft. **Giorgio Compagnucci:** investigation, resources. **Andrea Abate:** data curation, formal analysis, writing – original draft. **Alessandro Ugolini:** writing – review and editing, supervision. **Valentina Lanteri:** writing – review and editing, supervision.

## Funding

No funding was received for this manuscript.

## Disclosure

All authors have read and approved the final version of the manuscript. Alessandro Bruni had full access to all of the data in this study and takes complete responsibility for the integrity of the data and the accuracy of the data analysis.

## Ethics Statement

This study involved the management of individual clinical cases and did not require approval from an institutional ethics committee according to local regulations. All procedures were conducted in accordance with the ethical standards of the Declaration of Helsinki.

## Consent

Written informed consent was obtained from all patients for the treatment and for the publication of anonymized clinical data and images.

## Conflicts of Interest

The authors declare no conflicts of interest.

## Data Availability

Data sharing not applicable to this article as no datasets were generated or analyzed during the current study.
